# Effects of physical activity intervention on 24-h movement behaviors: a compositional data analysis

**DOI:** 10.1038/s41598-022-12715-2

**Published:** 2022-05-24

**Authors:** Jesse Pasanen, Tuija Leskinen, Kristin Suorsa, Anna Pulakka, Joni Virta, Kari Auranen, Sari Stenholm

**Affiliations:** 1 Department of Public Health, University of Turku and Turku University Hospital, 20014 Turku, Finland; 2 Centre for Population Health Research, University of Turku and Turku University Hospital, Turku, Finland; 3 Finnish Institute for Health and Welfare, Helsinki, Finland; 4 Faculty of Medicine, Center for Life Course Health Research, University of Oulu, Oulu, Finland; 5 Department of Mathematics and Statistics, University of Turku, Turku, Finland; 6 Department of Clinical Medicine, University of Turku, Turku, Finland

**Keywords:** Applied mathematics, Lifestyle modification, Epidemiology

## Abstract

We utilized compositional data analysis (CoDA) to study changes in the composition of the 24-h movement behaviors during an activity tracker based physical activity intervention. A total of 231 recently retired Finnish retirees were randomized into intervention and control groups. The intervention participants were requested to use a commercial activity tracker bracelet with daily activity goal and inactivity alerts for 12 months. The controls received no intervention. The 24-h movement behaviors, i.e., sleep, sedentary time (SED), light physical activity (LPA), and moderate-to-vigorous physical activity (MVPA) were estimated from wrist-worn ActiGraph data using the GGIR R-package. Three balance coordinates describing the composition of movement behaviors were applied: ratio of active vs. passive behaviors, LPA vs. MVPA, and sleep vs. SED. A linear mixed model was used to study changes between the baseline and 6-month time point. Overall, the changes in the 24-h movement behaviors were small and did not differ between the groups. Only the ratio of LPA to MVPA tended to change differently between the groups (group*time interaction p = 0.08) as the intervention group increased LPA similarly to controls but decreased their MVPA. In conclusion, the use of a commercial activity tracker may not be enough to induce changes in the 24-h movement behaviors among retirees.

## Introduction

Traditionally, movement behaviors, i.e., physical activity, sedentary behavior and sleep have been studied in isolation from each other. However, when studying movement behaviors in isolation, one neglects the fact that increasing one type of behavior inevitably decreases the portion of at least one of the other types as the total time spent in different movement behaviors is fixed to 24 h per day^1^. Therefore, it has been suggested that all movement behaviors should be treated as a one 24-h composition, consisting of the proportions of the different types of activities within the composition^2,3^.

Compositional data analysis^4^ (CoDA) has been established as a suitable family of statistical methods to study 24-h movement behavior composition^2,3^. There are already studies which have examined the associations between 24-h movement behavior composition and different health outcomes in children^5–7^ and adults^3,8–13^ in cross-sectional or prospective study designs. In addition, recommendations of the optimal 24-h composition of the movement behaviors have been given^14,15^. However, studies examining the effect of an intervention on the change in the 24-h movement behavior composition over time are still scarce.

One previous randomized controlled trial (RCT) applied CoDA to study the effect of a workplace sitting-reduction intervention on wake-time sitting, standing and moving, and reported significant changes on work-time sitting and standing time (− 110 min and + 108 min per 16-h day, respectively) but not on leisure-time behavior^16^. In an another RCT study, two types of interventions, one aiming at decreasing sedentary time and another aiming at increasing physical activity, were conducted among office workers^17^, but no intervention effects on 24-h movement behaviors were found. These previous RCTs thus suggest that interventions targeted to workplace or aiming to change only one type of movement behavior may be inadequate to change the 24-h composition.

In the present study, we applied the CoDA methodology to examine changes in 24-h movement behaviors during a physical activity intervention that aimed at both increasing daily physical activity and decreasing sedentary time among Finnish retirees with a daily use of a commercial activity tracker (REACT trial, NCT03320746). The effect of the activity tracker based intervention on accelerometer-measured total physical activity^18^ and sedentary time^19^ over 12 months have previously been reported. These separately analyzed results showed no intervention effect on total physical activity or sedentary time over the 12 months but a trend towards increased light physical activity and decreased prolonged sedentary time over the first 6 months^18,19^. The aim of the present study is utilize CoDA methods to obtain a more comprehensive understanding of the changes in all movement behaviors simultaneously during the first 6 months of the intervention.

## Results

Table 1 presents the baseline characteristics of the 231 study participants, randomized to the intervention and control groups. The mean age of the participants was 65.2 years (SD 1.1) and 83% of them were women. The intervention and control groups did not differ in terms of occupational status, body mass index, or the number of chronic diseases at baseline. Table 2 shows the compositional means of the time-use in each movement behavior for the intervention and control groups at each follow-up time point (baseline, 3 months, 6 months and 12 months). At baseline, the participants spent on average over 11 h per day for sedentary activities (SED), 8 h for sleeping, 4 h in light physical activity (LPA), and 40 to 50 min in moderate-to-vigorous physical activity (MVPA). Supplementary Fig. S1 illustrates the time-use in each movement behavior at baseline and the 6-month time point, the two time points of interest in the present study, in four ternary plots. Each plot shows the composition as a three-dimensional sub-composition. There appears to be a small shift towards increased time-use in LPA in both groups.

**Table 1:** Baseline characteristics of the intervention and the control groups.

Characteristic	Intervention (n = 117)	Control (n = 114)
	Mean (SD)	Mean (SD)
Age, years	65.2 (1.0)	65.2 (1.1)

	n (%)	n (%)
**Gender**		
Women	96 (82.0)	95 (83.3)
Men	21 (18.0)	19 (16.7)
**Occupational status**		
High	47 (40.2)	41 (36.0)
Intermediate	35 (29.9)	28 (24.5)
Low	35 (29.9)	45 (39.5)
**Body mass index**		
Under/normal weight	38 (32.5)	43 (37.7)
Overweight	43 (36.7)	45 (39.5)
Obese	36 (30.7)	26 (22.8)
**Number of chronic conditions**		
0	36 (30.8)	27 (23.9)
1	47 (40.2)	45 (49.8)
> 1	34 (29.1)	41 (36.3)

High occupational status includes managers and professionals, intermediate includes associate professionals, and low includes manual and service workers by the last known occupation preceding the retirement. Body mass index (BMI) is calculated from the measured height and weight. Chronic conditions include angina pectoris, myocardial infarction, stroke, claudication, osteoarthritis, osteoporosis, sciatica, fibromyalgia, rheumatoid arthritis, diabetes, depression or other mental illness.

**Table 2:** Mean time-use of movement behaviors in the intervention and the control groups at the four time points of the trial.

Time point	Control				Intervention			
	SED min	LPA min	MVPA min	Sleep min	SED min	LPA min	MVPA min	Sleep min
Baseline	677.5	217.3	42.5	502.7	670.8	224.3	51.5	493.5
3 months	661.0	233.7	43.9	501.4	669.6	231.5	50.3	488.5
6 months	664.6	235.1	42.5	497.8	658.9	246.4	43.6	491.1
12 months	684.9	215.9	39.9	499.3	681.0	210.6	46.5	501.9

The means are scaled to sum up to 1440 min (24 h).

*SED* sedentary behavior, *LPA* light physical activity, *MVPA* moderate-to-vigorous physical activity.

**Figure 1:**
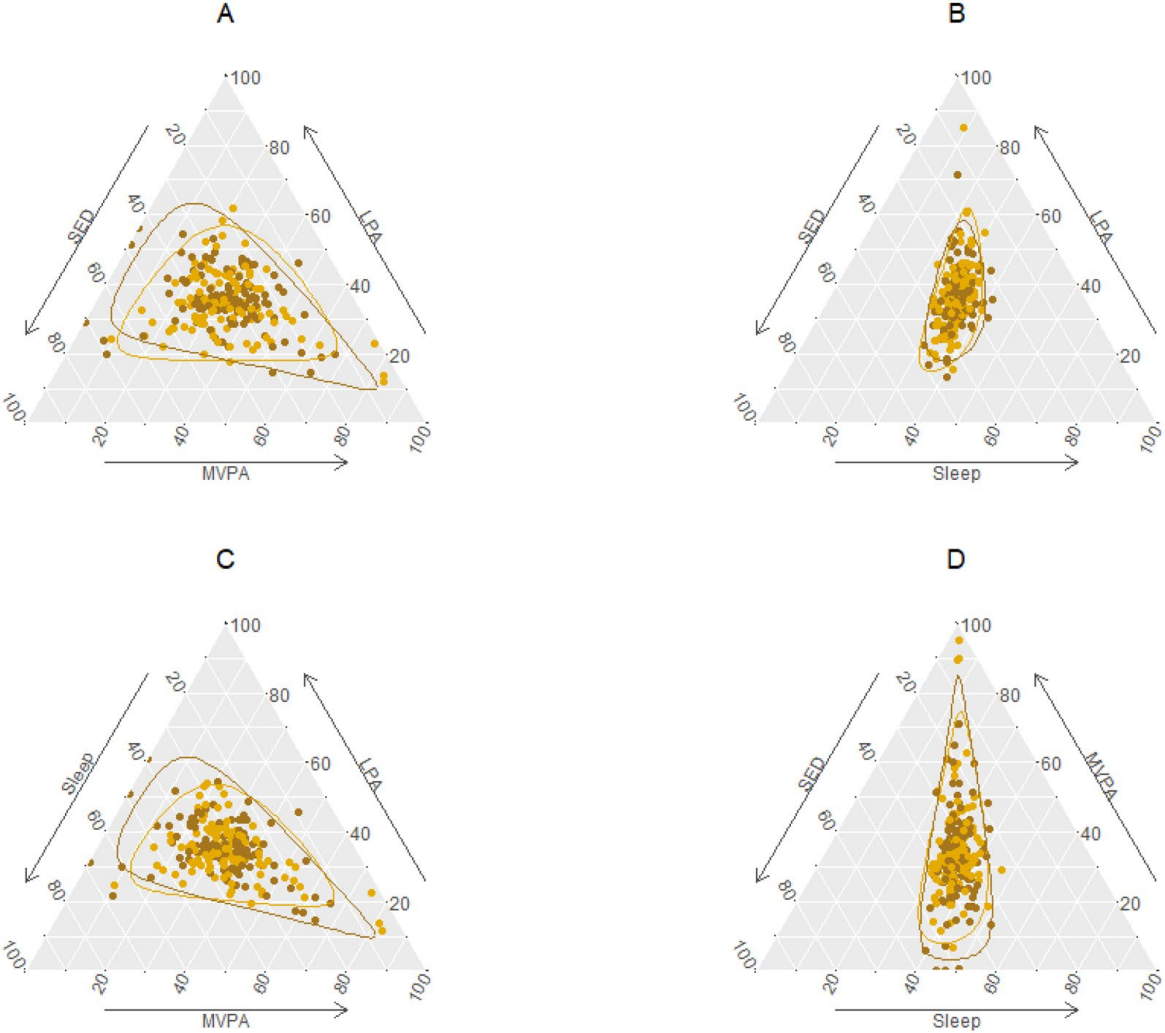
Ternary plots of the compositions of the compositional differences between the baseline and 6-month time point and the 95% confidence regions for the control (yellow dots and line) and the intervention (brown dots and line) group participants. Each plot (**A**–**D**) shows the composition as a three-dimensional sub-composition. The center of the plot corresponds to having no change in movement behaviors. The 95% confidence regions are based on an assumption of normality. *SED* sedentary behavior, *LPA* light physical activity, *MVPA* moderate-to-vigorous physical activity.

To further examine the changes in movement behaviors over the first 6 months of the trial, the compositional differences between the baseline and the 6-month time point were calculated by perturbing (i.e., dividing component-wise) the 6-months’ composition with the baseline composition, resulting in compositions which indicate how much the ratios between different sub-components changed over time for each individual subject. Figure 1 presents the ternary plots of the composition of the compositional differences and the 95% confidence regions for the intervention and control groups. Supplementary Fig. S2 illustrates an example on how to interpret the plots of the changes in movement behaviors. Overall, the majority of the observations of the change lied close to the centers of the four plots thus indicating that the ratios between the corresponding sub-components did not markedly change over time (Fig. 1). These descriptive findings show that in both groups the change (increase and decrease) in MVPA (Fig. 1D) was more pronounced than the change in LPA (Fig. 1B), whereas the ratio between SED and sleep remained more or less the same. The 95% confidence regions indicate that the change in the proportion of MVPA is somewhat larger for the intervention group compared to the control group (Fig. 1A,C,D).

For the CoDA analysis, each compositional part was assigned as either negative or positive in order to formulate balance coordinates, a specific type of isometric logratio coordinates (see Supplementary Table S1 for details). Overall, three balance coordinates were used: the ratio of active (including LPA and MVPA) vs. passive (including SED and sleep) movement behaviors (balance coordinate 1), the ratio of LPA vs. MVPA (balance coordinate 2), and the ratio of SED vs. sleep (balance coordinate 3). The changes in all three balance coordinates over the 6 months were then compared between the groups using linear mixed models, with random effects accounting for heterogeneity across individuals.

The change in the ratio of active vs. passive movement behaviors and the change in the ratio of SED vs. sleep was not different between the groups over the 6 months (group*time interaction p = 0.34 and p = 0.64, respectively) (Table 3). However, there was a borderline interaction for the ratio of LPA vs. MVPA (group*time interaction p = 0.08) suggesting that the ratio of LPA vs. MVPA tended to increase more among the intervention compared to the control group.

**Table 3:** Summary of the estimates of the fixed effects and their 95% confidence intervals for the linear mixed models of the three balance coordinates vs. the covariates.

	β	95% CI		P-value
**Coordinate 1 (active vs. passive movement behaviors)**				
(Intercept)	− 1.82	− 2.00	− 1.65	0
Gender	0.06	− 0.11	0.22	0.5
Age	− 0.02	− 0.15	0.1	0.75
Group	0.12	− 0.01	0.26	0.08
Time	0.01	− 0.01	0.03	0.32
Group*Time	− 0.01	− 0.04	0.01	0.34
**Coordinate 2 (LPA vs. MVPA)**				
(Intercept)	1	0.88	1.12	0
Gender	0.12	0.01	0.24	0.03
Age	0.08	− 0.01	0.17	0.07
Group	− 0.13	− 0.22	− 0.03	0.01
Time	0.01	− 0.01	0.03	0.31
Group*Time	0.02	0	0.05	0.08
**Coordinate 3 (SED vs. sleep)**				
(Intercept)	0.24	0.19	0.29	0
Gender	− 0.06	− 0.11	− 0.01	0.02
Age	0.03	− 0.01	0.07	0.14
Group	0.01	− 0.03	0.05	0.52
Time	0	0	0	0.75
Group*Time	0	− 0.01	0	0.64

Group indicates intervention and control groups; Time term indicates change from baseline to 6-month time point.

*SED* sedentary behavior, *LPA* light physical activity, *MVPA* moderate-to-vigorous physical activity.

Because three of the observations in the dataset had MVPA values less than 0.5 min, we conducted a sensitivity analysis in which the near-to-zero MVPA observations were imputed close to a one minute value. The imputed data are shown as ternary plots of the compositions of the compositional differences in Supplementary Fig. S3. In the regression analysis, the results remained the same, except the interaction for the balance coordinate 2 weakened slightly (time*group interaction p = 0.12) (Supplementary Table S2).

## Discussion

This study examined the in-depth changes in the 24-h movement behavior composition during a commercial activity tracker based intervention, which aimed at increasing daily physical activity and decreasing sedentary time among Finnish retirees. The present study is among the first to utilize compositional data analysis (CoDA) to study changes in the 24-h movement behavior composition in an RCT setting. The CoDA findings extended our previous isolated analyses on physical activity and sedentary behavior^18,19^ to cover changes in all four movement behaviors (LPA, MVPA, SED and sleep) simultaneously. The results showed that the changes in the 24-h movement behaviors during the activity tracker based intervention were rather small and not notably different from that of the controls.

Our findings are in line with the previous RCT study by Larisch et al.^17^ utilizing CoDA to evaluate the effects of two types of interventions, one aimed at increasing physical activity and another aimed at decreasing sedentary time among office workers. After 6 months, they found no intervention effect on the 24-h composition for either of the interventions^17^. In another RCT, the CoDA was used to examine the effect of a workplace sitting-reduction intervention on the replacement of sitting with standing during wake-time, with preferable changes found during work-time, but not during leisure time^16^. Compared to these previous intervention studies, our study evaluated a more comprehensive intervention targeted to change all wake-time movement behaviors, and included all movement behaviors to the 24-h composition. In addition, we utilized balance coordinates to compare e.g., active behaviors to passive behaviors, which we observed to be a more sensitive way of detecting shifting towards more activity at the cost of passive behaviors, when compared to pivot coordinates comparing one component at a time to all the remaining components^17^. However, despite the potentially more appropriate approach, the activity tracker based intervention was not efficient enough to elicit changes to the 24-h composition.

According to a recent systematic review, reallocating time to MVPA is associated with the most favorable health outcomes^12^. Our compositional data showed that only few of the intervention participants did increase MVPA over time and that there were also participants who replaced MVPA with LPA, a phenomenon that was neither expected nor wanted, and also weakened the effect at the group level. Consequently, the borderline difference in the change of the LPA vs. MVPA ratio between the intervention and control groups was seen as the intervention group increased LPA similarly to controls but they simultaneously decreased MVPA. The increase in the LPA component among the control group participants could be due to the baseline measurements, including e.g., body composition measure, which may have motivated them to increase physical activity. In addition, follow-up measurements with accelerometers may have increased physical activity among the control group participants, although the accelerometers did not give any feedback to the users.

The commercial activity tracker used as a stand-alone intervention method in our trial had two main features, described in detail elsewhere^18^. Briefly, the daily activity goal was displayed on the screen of the tracker and it was accompanied with a guidance on how to achieve the daily goal, for example “jog for 30 min” (goal for MVPA) or “walk for 50 min” (goal for LPA). The tracker also prompted the user to break up a non-movement period after 55 min with an inactivity alert, including vibration and a text “it’s time to move” on the screen of the bracelet. The fact that the user was able to reach the daily activity goal by accumulating activities with different intensities, including daily chores, may have resulted in both expected and unexpected changes in wake-time movement behaviors, i.e., the increase in LPA at the cost of SED and/or MVPA. The lack of a larger effect on replacing SED with physical activity, thus including the failure to increase MVPA, may be regarded as the main pitfall of our intervention. Therefore, future studies should investigate whether other intervention methods, preferably targeted to increase both types of daily physical activity would lead to more marked changes in the 24-h composition. A recommendation of this type of behavioral change is included in the current physical activity guidelines^20^.

The current study has several strengths but also some limitations, which need to be acknowledged. The participants’ adherence to the follow-up measurements was excellent, and the use of the activity trackers among the intervention participants was followed via a web-based program throughout the intervention^18^. The use of wrist-worn accelerometers for 24 h over eight days and seven nights enabled capturing all movement behaviors at different time points and thus utilizing the CoDA method to study changes in the 24-h composition over time. As a limitation, the threshold values used in the GGIR software to identify SED, LPA and MVPA were developed in small study populations of younger adults, among 20-year-olds (n = 20)^21^ and 30-year-olds (n = 30)^22^. Moreover, it should be acknowledged that there may be some inaccuracy in the absolute values of movement behaviors when using wrist-worn accelerometers. In sitting postures with arm movements, some sedentary behaviors may be misclassified as physical activity and on the other hand, standing and physical activity with no or little arm movements may be misclassified as sedentary behavior. Thus, wrist-worn accelerometers, when processed with GGIR, tend to overestimate sedentary time when compared with posture-based methods in free-living conditions^23^. The threshold values used for LPA and MVPA have been reported to have reasonable accuracy when compared with direct observation in semi-standardized conditions^21^ and with indirect calorimetry in free-living conditions^22^. However, our aim was to examine and compare intra-individual changes rather than absolute values of movement behaviors over time between the intervention and control groups. Overestimation of sedentary time is unlikely as critical for the changes in movement behaviors as it is for studying the absolute values. In addition, although there was no particular reason to suspect measurement errors for values of MVPA under 0.5 min, we conducted a sensitivity analysis with the imputed data, because CoDA may be affected by the near-to-zero values. The imputed data showed results similar to the main analysis. The sample size of the study was calculated for the physical activity component only and may therefore be underpowered for the secondary analyses. Finally, the majority of the study population were active and healthy women which may limit the generalizability of the findings.

In conclusion, the CoDA findings showed that the daily use of commercial activity trackers had no effect on the 24-h movement behavior composition over the 6 months among general population of recent retirees, when it was compared to the non-user controls. Future interventions aiming to change the 24-h movement behavior composition should use complementary approaches to target preferably all movement behaviors simultaneously.

## Methods

### Study design

The study followed the guidelines of good scientific practice set by the National Advisory Board on Research Ethics in Finland and the Declaration of Helsinki. The ethics committee of the Hospital District of Southwest Finland (107/1801/2017) has approved the REACT trial (NCT03320746, 25/10/2017). All participants were informed of the study protocol and their voluntariness before expressing their willingness to participate and giving a signed, informed consent. The inclusion criteria for the REACT trial were: self-reported actual date of retirement between January 2016 and December 2018, self-reported ability to walk 500 m without interruption, no current post-operative state, no known surgery within the next 6 months, no malign cancer or recent myocardial infraction, basic knowledge on how to use a computer, and Internet access at home.

The recruitment, randomization, and the intervention protocol have been reported in detail elsewhere^18^. Briefly, the study population of the REACT trial included 231 Finnish public sector employees who had retired between January 2016 and December 2018. The participants were randomized into two groups with an allocation ratio of 1:1 and using randomization lists for men and women separately. The participants randomized to the intervention group (n = 117) were requested to wear a commercial activity tracker (Polar Loop 2, Polar, Kempele, Finland) on the non-dominant wrist every day and night for 12 months and to aim to reach the daily activity goal shown on the tracker’s display. The initial daily activity goal was set to correspond to for example ~ 1 h/day of jogging, or ~ 2 h/day of walking, or ~ 7 h/day of household activities, or a combination of these activities. Since the Polar Loop 2 is tracking activity with a built-in accelerometer, various kinds of activities could contribute to the achievement of the daily activity goal so that activities at higher intensities helped to reach the daily goal faster than activities at lower intensities. During each day, the tracker provided an instant view on how much activity was still needed to reach the goal and gave an inactivity alert “it’s time to move” every time the user had an uninterrupted non-movement time for 55 min. A higher daily activity goal was suggested by the researcher if the participant frequently exceeded the former goal. No further guidance on how to achieve the daily goal was given. The Polar’s web-based program (Polar Flow) was used to upload the individual daily activity measurements and to provide the user more detailed feedback on the health benefits of the accumulated activity, sedentary time, and sleep. The control group members (n = 114) were requested to abstain from the use of any type of activity trackers during the 12-month follow-up.

### Measurement of the 24-h movement behavior

The 24-h movement behaviors, namely sleep, sedentary time (SED), light physical activity (LPA) and moderate-to-vigorous physical activity (MVPA), were measured using a wrist-worn triaxial ActiGraph wGT3X-BT. The participants wore the device on their non-dominant wrist for eight days and seven nights at baseline, 3 months, 6 months and 12 months’ time points^18^. The accelerometer data were processed to daily estimates of 24-h movement behaviors using the R-package GGIR version 1.7-1^24^, which is a highly used tool in the field (https://github.com/wadpac/GGIR/wiki/Publication-list) and a suitable software to process wrist-worn accelerometer data to daily estimates of the 24-h movement behaviors. Categorization of movement behaviors is based on thresholds i.e., ranges in acceleration that have been developed in methodological studies^21,22^. The GGIR allows researchers to make different data reduction choices and to choose the threshold values for movement behaviors. Our data reduction choices can be seen in Supplementary File S1. Accelerometer wear time was detected between the first and last recorded times in the daily log and non-wear time was excluded following the principles in the GGIR^25^. The GGIR classified non-wear time using 15 min time blocks based on the characteristics of the 60 min time window centered at these 15 min. A block was classified as non-wear time if the standard deviation of the 60 min window was less than 13.0 mg (milli gravity-based acceleration unit, where 1 g = 9.81 m/s^2^) for at least two out of the three axes or if the value range, for at least two out of three axes, was less than 50 m*g*^25,26^. Sleep time was estimated using the sleep algorithm in the GGIR^27^ and daily logs, so that sleep was defined as periods of time within the bedtime and waking times reported in the daily logs during which there was no change larger than 5° in the arm angle over at least 5 min. Time spent in LPA and MVPA were determined using previously proposed threshold values^21,22^, ≥ 30.0 mg for LPA and ≥ 100.6 mg for MVPA. SED was defined using a previously proposed threshold of < 30.0 m*g*^21^.

For the analyses, we defined one day to constitute of the time period from one measurement day’s in-bed time to the next measurement day’s in-bed time. The average duration of each measurement day was approximately 1412 min (95% CI 1408–1416) at baseline and 1425 min (95% CI 1423–1427) at the 6-month time point. The analyses were restricted to valid measurement days with at least 10 h of waking wear time of the accelerometer. Further, only those study participants who had at least four valid measurement days at both baseline and the 6-month time point, were included, leading to exclusion of seven study participants.

### Statistical analysis

Participant characteristics were described using means and standard deviations for continuous variables and frequencies and percentages for categorical variables. All statistical analyses were performed in the R software version 4.0.3 (R Foundation for Statistical Computing, Vienna, Austria).

Compositional analyses were conducted using the packages compositions^28^, robCompositions^29^, and nlme^30^. The compositional data included three missing values and one zero value, which were imputed using the package robCompositions before any compositional data analysis methods were used. Missing values were imputed with the function impCoda(), while the zero value was imputed with the function impRZilr().

### Descriptive analysis of compositional data

Compositional means were calculated as component-wise geometric means of the observed time use, and rescaled to sum up to 1440 min. Ternary plots were drawn to visualize the relationships between the different sub-compositions of the full composition using the package ggtern. The plots were drawn in groups of four, with one plot for each possible three-dimensional sub-composition of the four-part composition. Confidence regions were calculated with ggtern and they were based on a normality assumption and the Mahalanobis distance of the logratio transformation.

The compositional differences between the baseline and the 6-month compositions were calculated for each participant via perturbation, which is a compositional operation analogous to addition or subtraction. In practice, this meant scaling each observation so that the sum of its compositional parts was one (1), and then dividing each part of the 6-month observation with the corresponding part of the baseline observation. The resulting composition of the compositional differences is visualized as ternary plots.

### Compositional data analysis

An isometric logratio (ilr) transformation was used to map the compositional observations to real-valued coordinates, thus reducing the dimensionality of the data and allowing standard statistical methods to be used^4^. The specific type of ilr coordinates used in this study were *balance coordinates*. Compared to other commonly-used ilr coordinates, such as pivot coordinates which focus on one part out of the whole composition, balance coordinates can be used to flexibly study the relationships between groups of parts, giving a broader overview of the entire composition. The balance coordinates were formed by assigning compositional parts into opposing groups, with each coordinate pertaining to a positive or negative group. These groups were then used to calculate the coordinates in such a way that each coordinate represents the ratio of the sizes of its groups, in other words how much larger the combined proportional size of the parts in one group is compared to the parts in the other group.

The groups of compositional parts for each coordinate were chosen using sequential binary partitioning (Supplementary Table S1). First, LPA and MVPA were selected as positive, and sleep and sedentary behaviors were selected as negative (balance coordinate 1). These sub-compositions correspond to the first coordinate of the transformation, with positive values of the coordinate indicating that the positive group is dominant and vice versa. For the second coordinate, the parts of the previous positive sub-composition were divided, with LPA selected as positive and MVPA selected as negative (balance coordinate 2). Thus, positive values of the second coordinate corresponded to LPA being dominant in the ratio of LPA vs. MVPA. Finally, for the third coordinate, the negative sub-composition was divided, with positive values corresponding to the dominance of SED in the ratio of SED vs. sleep (balance coordinate 3).

After the binary partition was selected, the balance coordinates were calculated using the formula^31^

$${ilr}_{k}=\sqrt{\frac{{p}_{k}{m}_{k}}{{p}_{k}+{m}_{k}}}\mathrm{ln}\frac{{\left({x}_{{i}_{1}}{x}_{{i}_{2}}\cdots {x}_{{i}_{{p}_{k}}}\right)}^{\frac{1}{{p}_{k}}}}{{\left({y}_{{j}_{1}}{y}_{{j}_{2}}\cdots {y}_{{j}_{{m}_{k}}}\right)}^{\frac{1}{{m}_{k}}}}$$

where ${p}_{k}$ and ${m}_{k}$ are the number of compositional parts in positive and negative groups of coordinate ${z}_{k}$, and ${x}_{i}$ and ${y}_{j}$ are the proportions of each part in these groups. With the binary partition used in this article, the equations become fairly simple, and the balance coordinates for each observation were calculated as follows:

$${ilr}_{1}=\mathrm{ln}\frac{\sqrt{LPA*MVPA}}{\sqrt{SED*SLEEP}}$$

$${ilr}_{2}=\sqrt{\frac{1}{2}} \mathrm{ln }\frac{LPA}{MVPA}$$

$${ilr}_{3}=\sqrt{\frac{1}{2}} \mathrm{ ln} \frac{SED}{SLEEP}$$

where LPA, MVPA, SED and SLEEP refer to the proportions of these parts in the observation, corresponding to ${x}_{i}$ and ${y}_{j}$ in the above formula.

Of note is that the second coordinate contains information only about the relationship between LPA and MVPA and not about their relationship to the remaining two components and, similarly, the third coordinate contains information only of the relationship between SED and sleep.

After the coordinate transformation was applied to the compositional data, a separate linear mixed model was fitted for each of the three coordinates. Linear mixed models were used to study the changes in the movement behaviors over the 6-month follow-up. The fixed effects of each model were the main effects of time, age, sex, group, and the interaction time*group. In all three models, subject-specific random effects were applied in terms of a random intercept and a random slope of time.

Three observations in the dataset had MVPA values less than 0.5 min, including the one zero value, which was imputed to the original composition data. There was no particular reason to suspect measurement error for values under 0.5 min of MVPA, but values close to zero can skew results of compositional data analysis in subtle ways. To counteract this, we conducted a sensitivity analysis in which the observations under 0.5 min were imputed using the same method as for the missing values and zeros. The imputation increased those MVPA values close to one minute. The linear mixed analysis was repeated for the imputed dataset.

## Supplementary Information

Supplementary Information.

## Data Availability

Pseudonymized partial data sets of the REACT trial are available by application with bona fide researchers with an established scientific record and bona fide organizations.
