# Supplementary material for: Effects of physical activity intervention on 24-h movement behaviors: a compositional data analysis
Source: Sci Rep. 2022 May 24;12:8712. doi: 10.1038/s41598-022-12715-2 (PMC9130120; doi:10.1038/s41598-022-12715-2)
Supplement: Supplementary file 1 — Supplementary Information. [file 41598_2022_12715_MOESM1_ESM.docx]

**Effects of physical activity intervention on 24-hour movement behaviors: A compositional data analysis**

Jesse Pasanen 1*, Tuija Leskinen 1,2*, Kristin Suorsa 1,2, Anna Pulakka, 3,4, Joni Virta 5, Kari Auranen 5,6 Sari Stenholm 1,2

1 Department of Public Health, University of Turku and Turku University Hospital, Finland

2 Centre for Population Health Research, University of Turku and Turku University Hospital, Finland

3 Finnish Institute for Health and Welfare, Helsinki, Finland

4 Center for Life Course Health Research, Faculty of Medicine, University of Oulu, Oulu, Finland

5 Department of Mathematics and Statistics, University of Turku

6 Department of Clinical Medicine, University of Turku, Finland

*equal contribution

**Correspondence:** Dr. Tuija Leskinen, Department of Public Health, FI-20014 University of Turku, Finland, phone: +35823338440, fax: +358294505040, email: tuija.leskinen@utu.fi

**Figure S1. Ternary plots of the time-use of the movement behaviors for the intervention and control group participants at baseline and 6-month time point.** Each plot (A, B, C, and D) shows the composition as a three-dimensional sub-composition. The position of an observation on a plot indicates the ratio of the corresponding three components. The compositional means and their 95% confidence regions are presented for control (red dot and line) and intervention (green triangle and line) groups at baseline, and for control (blue square and line) and intervention (purple plus sign and line) groups at 6 months.


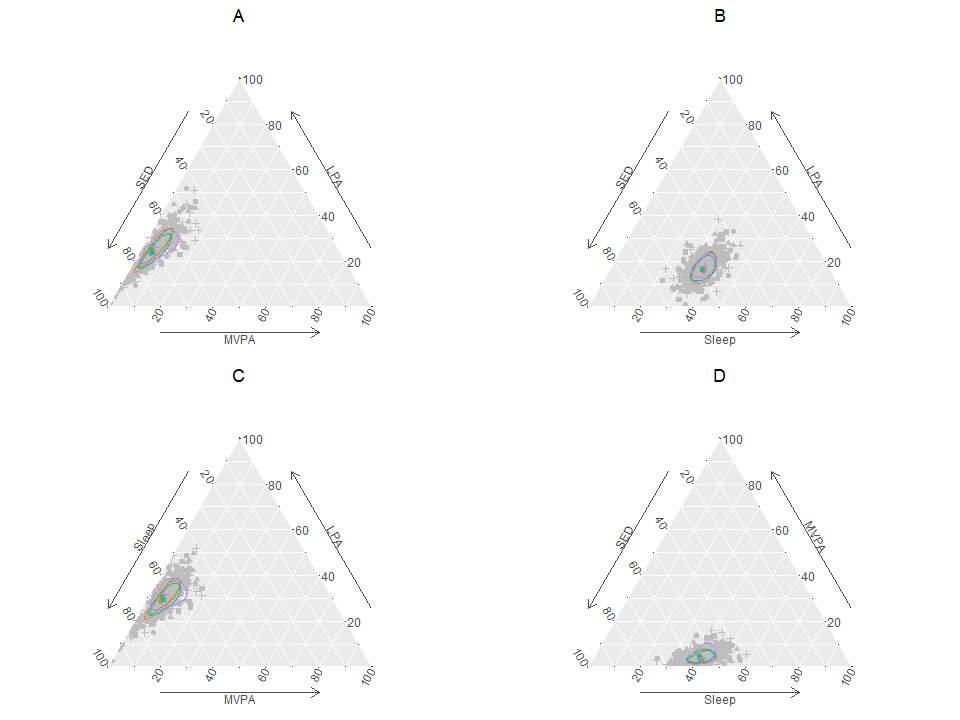


SED=sedentary behavior, LPA=light physical activity, MVPA=moderate-to-vigorous physical activity

**Interpretation of Figure S1:** Ternary plots can be used to describe compositions with more than three parts, as in this case when the 24-hour movement behavior has four parts. Each plot represents a three-part subset of the whole 24-hour movement behavior composition. Each dot corresponds to an observation and describes the ratio between the three components. For any single dot, the three readings, one from each scale, sum up to 100. Dots near the center, where each scale’s 33% lines meet, represent compositions where the three components are approximately equal. Dots close to a corner represent compositions where one of the components is much larger compared to the other two components. Each plot describes the relationship between the three parts, but not how these three parts are related to the one part which is not included in that plot. Therefore, multiple plots are needed to gain an understanding of the entire 24-hour composition.

**Figure S2. Example on how to interpret a ternary plot of the changes in movement behaviors over time.**


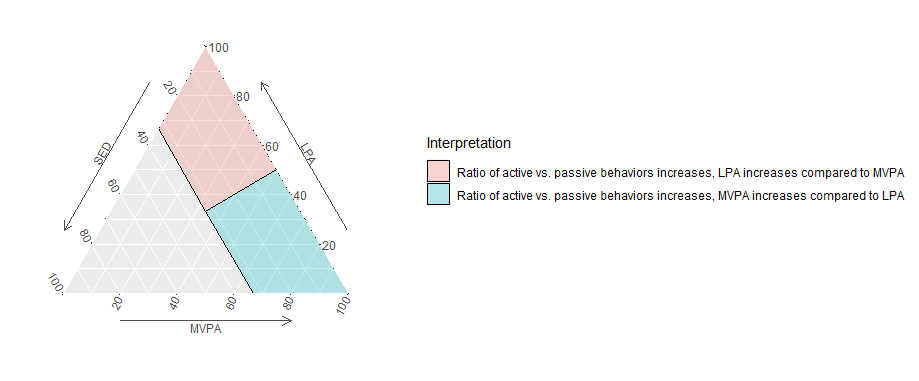


SED=sedentary behavior, LPA=light physical activity, MVPA=moderate-to-vigorous physical activity

**Interpretation of Figure S2:** Figure depicts compositional differences, i.e. shows how those proportions changed over time. An observation (usually a dot) close to the center of the plot indicates that the ratio between the corresponding parts did not change over time. Observations closer to the edges and corners of the plot indicate that the ratio between the parts have changed. The closer the observation is to a specific part’s corner, the more that part increased its ratio in comparison to the other parts on the graph. In the example graph, observations where the ratio of LPA and MPVA to SED increased would fall in the colored areas. More spesifically, those observations where the ratio of LPA to MVPA increased would fall in the upper red area, while those where the ratio of LPA to MVPA decreased would fall in the lower blue area.

**Figure S3. Ternary plots of the compositions of the compositional differences between the baseline and 6-month time point and the 95% confidence regions for the control (yellow dots and line) and the intervention (brown dots and line) group participants in the imputed data**. Each plot (A, B, C, and D) shows the composition as a three-dimensional sub-composition. The 95% confidence regions are based on an assumption of normality.


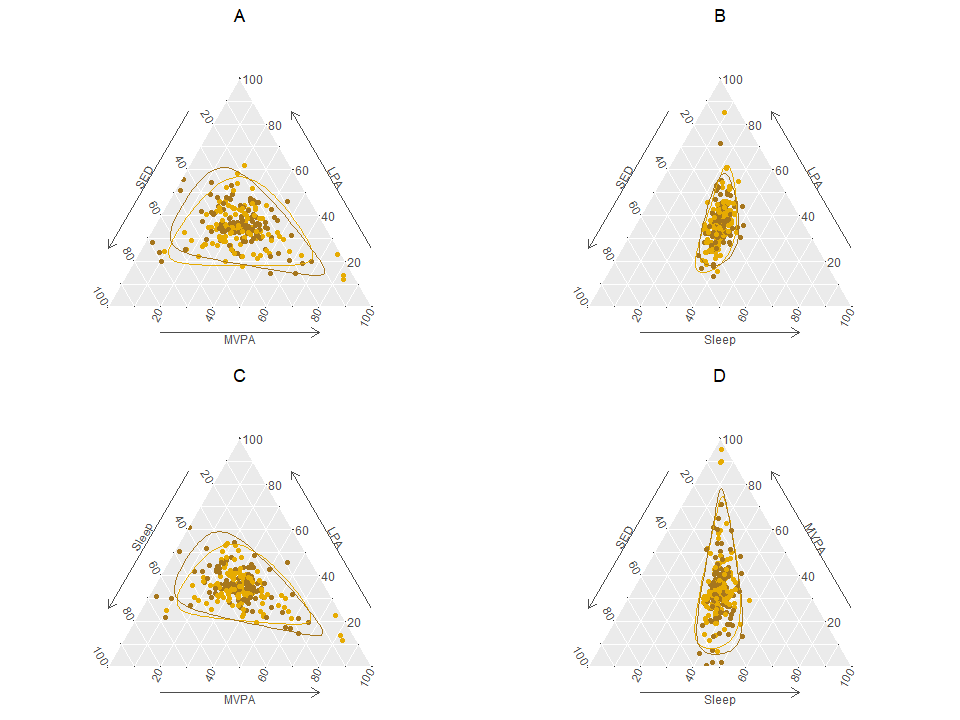


SED=sedentary behavior, LPA=light physical activity, MVPA=moderate-to-vigorous physical activity

**Table S1. The sequential binary partition used for the balance coordinate transformation.** Each column indicates which parts of the composition make up the corresponding coordinate. For parts with the value -1, a higher proportion in the composition corresponds to lower values of the coordinate. For parts with the value 1, a higher proportion in the composition corresponds to higher values of the coordinate. The value 0 indicates that the part is not included in the coordinate.

| **Coordinate** | **1** | **2** | **3** |
| --- | --- | --- | --- |
| SED | -1 | 0 | 1 |
| LPA | 1 | 1 | 0 |
| MVPA | 1 | -1 | 0 |
| Sleep | -1 | 0 | -1 |

SED=sedentary behavior, LPA=light physical activity, MVPA=moderate-to-vigorous physical activity

**Table S2.** **Summary of the estimates of the fixed effects and their 95% confidence intervals for the linear mixed models of the three balance coordinates vs. the covariates for the imputed dataset.** Coordinate 1 is for ratio of active vs. passive movement behaviors, coordinate 2 for ratio of LPA vs. MVPA, and coordinate 3 for ratio of SED vs. sleep. The time period is from baseline to 6 months.

|  | Value | 95 % CI | | P value |
| --- | --- | --- | --- | --- |
| Coordinate 1 |  |  |  |  |
| (Intercept) | -1.82 | -1.99 | -1.64 | 0 |
| sex | 0.05 | -0.11 | 0.21 | 0.52 |
| age | -0.04 | -0.15 | 0.09 | 0.65 |
| group | 0.12 | -0.02 | 0.26 | 0.09 |
| time | 0.01 | -0.01 | 0.03 | 0.28 |
| group*time | -0.01 | -0.03 | 0.01 | 0.47 |
|  |  |  |  |  |
| Coordinate 2 |  |  |  |  |
| (Intercept) | 1 | 0.89 | 1.12 | 0 |
| sex | 0.12 | 0.01 | 0.23 | 0.03 |
| age | 0.08 | -0.01 | 0.17 | 0.06 |
| group | -0.12 | -0.22 | -0.03 | 0.01 |
| time | 0.01 | -0.01 | 0.02 | 0.23 |
| group*time | 0.02 | 0 | 0.04 | 0.12 |
|  |  |  |  |  |
| Coordinate 3 |  |  |  |  |
| (Intercept) | 0.24 | 0.19 | 0.29 | 0 |
| sex | -0.06 | -0.11 | -0.01 | 0.02 |
| age | 0.03 | -0.01 | 0.07 | 0.14 |
| group | 0.01 | -0.03 | 0.05 | 0.52 |
| time | 0 | 0 | 0 | 0.75 |
| group*time | 0 | -0.01 | 0 | 0.64 |

**File S1.** The GGIR script.

library(GGIR)

f0=1

f1=231

g.shell.GGIR(#=======================================

mode=c(1,2,3,4,5),

datadir="/wrk/data",

outputdir="/wrk/results",

f0=f0, f1=f1,

daylimit=FALSE,

#-------------------------------

# Part 1:

#-------------------------------

windowsizes = c(5, 900, 3600),

desiredtz="Europe/Helsinki",

do.enmo = TRUE, do.anglez=TRUE,

chunksize=1, printsummary=TRUE,

overwrite=TRUE,

#-------------------------------

# Part 2:

#-------------------------------

strategy = 3,

ndayswindow=9,

winhr = c(5),

qwindow=c(0,24),

ilevels = c(seq(0,400,by=50),8000),

mvpathreshold =c(100.6),

bout.metric = 4,

epochvalues2csv=FALSE,

closedbout=FALSE,

do.imp = FALSE,

#-------------------------------

# Part 3:

#-------------------------------

# Key functions: Sleep detection

timethreshold= c(5), anglethreshold=5,

ignorenonwear = TRUE,

desiredtz="Europe/Helsinki",

#-------------------------------

# Part 4:

#-------------------------------

excludefirstlast = FALSE,

includenightcrit = 16,

def.noc.sleep = c(),

loglocation= c("/wrk/KL_log.csv"),

outliers.only = TRUE,

criterror = 4,

relyonsleeplog = FALSE,

sleeplogidnum = TRUE,

colid=1,

coln1=2,

do.visual = TRUE,

nnights = 9,

#-------------------------------

# Part 5:

# Key functions: Merging physical activity with sleep analyses

#-------------------------------

threshold.lig = c(30), threshold.mod = c(100.6), threshold.vig = c(428.8),

boutcriter = 0.8, boutcriter.in = 0.9, boutcriter.lig = 0.8,

boutcriter.mvpa = 0.8, boutdur.in = c(1,30,60), boutdur.lig = c(1,10),

boutdur.mvpa = c(1,10), timewindow = c("WW"), save_ms5rawlevels= TRUE,

#-----------------------------------

# Report generation

#-------------------------------

# Key functions: Generating reports based on meta-data

do.report=c(2,4,5),

visualreport=TRUE, dofirstpage = TRUE,

viewingwindow=1)
